# Lipid Droplets in Health and Disease

**DOI:** 10.1186/s12944-017-0521-7

**Published:** 2017-06-29

**Authors:** Gizem Onal, Ozlem Kutlu, Devrim Gozuacik, Serap Dokmeci Emre

**Affiliations:** 1 Department of Medical Biology, Hacettepe University, 06100 Ankara, Turkey; 2 Nanotechnology Research and Application Center (SUNUM) & Center of Excellence for Functional Surfaces and Interfaces for Nano Diagnostics (EFSUN), Sabanci University, 34956 Istanbul, Turkey; 3 Molecular Biology, Genetics, and Bioengineering Program & Center of Excellence for Functional Surfaces and Interfaces for Nano Diagnostics (EFSUN), Sabanci University, 34956 Istanbul, Turkey

**Keywords:** Lipid droplets, lipolysis, lipophagy, chaperone-mediated autophagy

## Abstract

Lipids are essential building blocks synthesized by complex molecular pathways and deposited as lipid droplets (LDs) in cells. LDs are evolutionary conserved organelles found in almost all organisms, from bacteria to mammals. They are composed of a hydrophobic neutral lipid core surrounding by a phospholipid monolayer membrane with various decorating proteins. Degradation of LDs provide metabolic energy for divergent cellular processes such as membrane synthesis and molecular signaling. Lipolysis and autophagy are two main catabolic pathways of LDs, which regulate lipid metabolism and, thereby, closely engaged in many pathological conditons. In this review, we first provide an overview of the current knowledge on the structural properties and the biogenesis of LDs. We further focus on the recent findings of their catabolic mechanism by lipolysis and autophagy as well as their connection ragarding the regulation and function. Moreover, we discuss the relevance of LDs and their catabolism-dependent pathophysiological conditions.

## Background

Many living organisms store lipids in their cells to produce metabolic energy, in case of insufficient energy sources. Cells preserve lipids by converting them into neutral lipids, such as triacylglycerides (TAG) and sterol esters (SE). These type of lipids are deposited in lipid droplets (LDs) which are also termed as adiposomes, lipid bodies or oil bodies [1]. For a long time, LDs were accepted as inert intracellular vesicles storing neutral lipids in all living organisms. However, recent advances in functional analysis techniques, imaging methods, lipidomics and proteomics technology offer scientists a better understanding of biological properties and functions of LDs. In the last couple of years, many structural and functional proteins were identified and characterized on the surface of LDs, and they were named as perilipins (PLINs) [2]. In addition to their role in energy metabolism, LDs play a role in various cellular events, ranging from protein degradation, sequestration of transcription factors and chromatin components to generation of lipid ligands for certain nuclear receptors, and they serve as fatty acid trafficking nodes [3–5]. Moreover, LDs might be hijacked by various pathogens. Due to these diverse functions, abnormalities of LDs were associated with many pathological conditions [6–8].

The catabolism of LDs into free fatty acids (FAs) is a crucial cellular pathway that is required to generate energy in the form of ATP, and to provide building blocks for biological membrane and hormone synthesis. Lipolysis is a biochemical catabolic pathway that relies on the direct activation of LD-associated lipases, such as adipose triglyceride lipase (ATGL), hormone-sensitive lipase (HSL) and monoglyceride lipase (MGL) [9, 10]. Together with regulatory protein factors ATGL activator (comperative gene identification-58, CGI-58) and ATGL inhibitor (G0/G1 switch protein 2, G0S2), these lipases constitute the basis for the lipolytic machinery in cells.

Autophagy is one of the two major degradation pathways in cells supporting cell survival by recycling metabolic components under stress conditions (Ubiquitin-proteasome system being the other pathway). It is initiated by sequestering cytosolic organelles or macromolecules in double-membrane vesicles and delivering them to lysosomes for degradation by the lytic enzymes therein. Cellular building blocks are then released back to cytosol and recycled to ensure cellular homeostasis [11, 12]. Basic machanism of autophagy is classified into three different types: Macroautophagy, chaperone-mediated autophagy (CMA) and microautophagy. Macroautophagy is a well-studied pathway, targeting large substrates such as toxic aggregates and degenerated organelles in a selective or non-selective manner [13]. Chaperone-mediated autophagy (CMA) is a selective form of autophagy, targeting specific proteins through the recognition activity of chaperone protein heat shock cognate 70 (Hsc70). Hsc70 delivers the substrates to the lysosomal lumen via lysosomal-associated protein 2a (LAMP2a) [14, 15]. Microautophagy degrades cytoplasmic cargos through a organized invagination of lysosomal membranes and direct engulfment [16].

Recent investigations defined another selective form of macroautophagy termed “lipophagy”. In this autophagy form, LD degradation is controlled by the association of membrane GTPase Rab7 with LDs [17, 18]. Moreover, a significant role of CMA was recently identified in selective degradation of a unique LD-coat protein family called PLINs [19, 20]. Therefore, autophagy pathways play a crucial role in lipid metabolism and the number of publications in this relatively new field is growing exponentially.

In contrast with the traditional view that LDs are simple lipid storage vesicles, because of their dynamic nature and multifunctionality they are today accepted as a distinct intracellular organelles. Recent studies underline physiological importance of these multifunctional organelles, particularly in the context of lipolysis and selective autophagy mechanisms of lipophagy and CMA. Here, we will first summarize biological properties of LDs and then focus on recent findings on molecular regulation of lipolytic and autophagic mechanism. Furthermore, we will discuss the relevance of LDs and related catabolic mechanisms for pathophysiology.

### Structure of the lipid droplets

#### Phospholipid composition

**Fig. 1:**
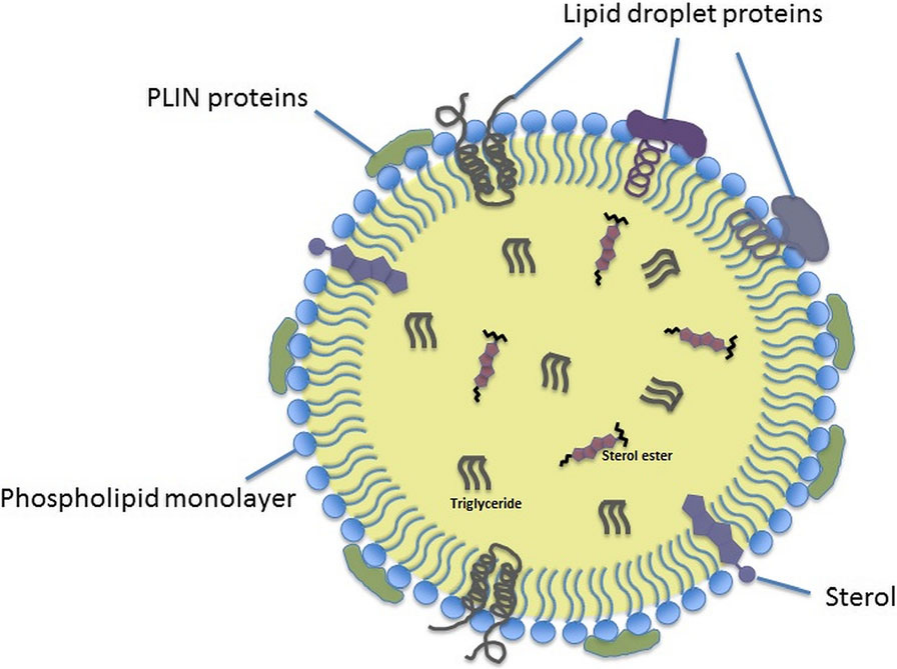
Basic Morphology of Lipid Droplets

Being different from other membrane-enclosed organelles, LDs have a unique structure with a hydrophobic core of neutral lipids surrounded by a monolayer phospholipid membrane, separating hydrophobic neutral lipids from the aqueous cytoplasmic environment [21].. In the hydrophobic core of LDs, neutral lipids, predominantly TG and SE, are stored at various ratios (Fig. 1). In white adipocytes, TG are primarily stored in LDs as lipid esters, whereas in steroidogenic cells, SE are the main components [22]. Also, depending on the cell type, many other endogenous neutral lipids such as retinyl esters, ether lipids, and free cholesterol are stored in LD cores [23–25]. Interestingly in some cell types, electron microscopy data revealed that membrane-like structures containing ribosomal units were also extending within the core of LDs [26, 27].

In mammalian cells, the main constituent (up to 60%) of LD monolayer membranes is phosphatidylcholine (PC) that is followed in abundance by phosphatidylethanolamine (PE), phosphatidylinositol (PI), phosphatidylserine (PS), sphingomyelin (SM) and lyso forms of PC and PE [28–30]. Phosphatidic acid and free cholesterol are also present in LD surfaces in minor amounts [21, 31]. Although LD formation was believed to take place between the leaflets of endoplasmic reticulum (ER), phospholipid composition of LD membrane differs from that of ER and other organelles [21]. The unique phospholipid membrane composition primarily affects LD synthesis [32], maturation (size) [33, 34] and degradation via lipophagy mechanisms [35, 36]. Changes in LD membrane phospholipid ratios under physiological conditions in various cell types indicate that regulation of phospholipid composition is crucial for homeostasis of this organelle.

#### Surface protein structure

Besides membrane phospholipid compositions, LD surface proteins are other factors regulating homeostasis and intracellular interactions of LDs. Membrane surface of LD is decorated with several structural and functional proteins [37]. Proteomics studies on LDs in a variety of organisms including bacteria [38–40], plants [41, 42], yeasts [43, 44], insects [45, 46] and mammals [47–49] revealed that over two hundred different proteins are localized to the surface of LDs. Even though LD proteomics studies that were performed in various cell types and organisms resulted in the discovery of different profiles, identified proteins seem to function in common pathways. These proteins were categorized into several distinct functional classes (Table 1).

**Table 1:** Classification of proteins located on the LD surface

Protein Group	Short Name	Long Name	References
PAT Family	PLIN	Perilipin	[49, 160]
	ADRP	Adipose differentiation-related protein	[24, 27, 48, 161–163]
	TIP47	Tail-interacting protein of 47 kD	[48, 160, 163–165]
Lipid & Energy Metabolism	HSL	Hormone sensitive lipase	[49, 160]
	ATGL (PNPLA2)	Adipose triglyceride lipase	[24, 162, 166]
	CGI-49	CGI-49 protein	[24, 27, 48, 49, 165]
	CGI-58	CGI-58 protein	[24, 27, 49, 160, 162]
	Mgll	Monoglyceride lipase	[97, 163]
	Tgh	Triacylglycerol hydrolase	[167]
	LACS3–4	Long-chain-fatty-acid–CoA ligase 3–4	[24, 48, 49, 162, 165]
	H105e3	Sterol-4-carboxylate 3-dehydrogenase	[48, 163–165]
	SE	Squalene monooxygenase	[27, 162, 168]
	Lss	Lanosterol synthase	[49, 160, 169]
	Cpla2	Cytosolic phospholipase A2	[24, 169, 170]
	Pcyt1a	Phosphocholine cytidylyltransferase A	[169, 171, 172]
	Mdh2	Malate dehydrogenase	[97, 173, 174]
	Cyb5r3	NADH-cytochrome b5 reductase	[24, 49, 162, 173]
	Dhrs1	Dehydrogenase/reductase SDR member 1	[24, 49, 162, 173]
	Dhrs3	Short-chain dehydrogenase/reductase 3	[92, 169, 173]
	Nsdhl	NAD(P)H steroid dehydrogenase-like	[163, 169, 173]
	Acsl1–3	Long-chain acyl-CoA synthetase 1–3	[24, 49, 175]
	Ldah	LD-associated hydrolase	[173, 176, 177]
Signalling	CHP	Calcium-binding protein p22	[24, 164, 165, 178]
	Cav1	Caveolin1	[24, 49, 162]
	METTL7A	Methyltransferase-like protein 7A	[92, 174, 178]
Membrane trafficking proteins	VIM	Vimentin	[24, 49, 160, 162, 171]
	ACTB	Actin, cytoplasmic 1/ □-Actin	[24, 27, 163, 165]
	Rab10	Ras-related protein Rab-10	[24, 27, 48, 162, 165]
	Rab 11A	Ras-related protein Rab-11A	[24, 162, 164]
	Rab 1a	Ras-related protein Rab-1a	[24, 162, 164, 165]
	Rab 1b	Ras-related protein Rab-1b	[48, 164, 165]
	Rab 14	Ras-related protein Rab-14	[24, 49, 160, 162, 164]
	Rab 18	Ras-related protein Rab-18	[24, 49, 162, 164, 165]
	Rab 5b	Ras-related protein Rab-5b	[24, 162, 165, 166]
	Tubulin	Tubulin	[24, 49, 163, 178]
Miscellaneous	Stomatin	Stomatin	[162, 165]
	HSPA5	78 kDa glucose- regulated protein	[24, 48, 49, 160, 162–165]
	Hspa1a	Heat shock 70 kDa protein 1A	[24, 49, 161]
	FAF2	FAS-associated factor 2	[24, 48, 162, 166]
	Ancient ubiquitous protein BiP	Ancient ubiquitous protein BiP	[24, 49, 160, 162, 165, 166]
	CANX	Calnexin	[49]
	HSP 70	Heat shock protein 70	[49, 161, 165]
	Ribophorin I	Ribophorin I	[49, 160]
	ApoB	Apolipoprotein B	[174]
	a-synuclein	a-synuclein	[92]
	Hepatitis C core protein	Hepatitis C core protein	[175–177]
	His2A	Histone 2A	[89]
	His2B	Histone 2B	[89]
	Alb	Serum albumin	[27, 161, 163]

In mammalian LDs, predominant proteins are the members of the PAT protein family, an acronym representing perilipin (PLIN), adipocyte differentiation-related protein (ADRP; also called as adipophilin), and TIP47 (tail-interacting protein of 47 kDa) proteins. Members of the PAT family share sequence similarity and the ability to bind intracellular LDs, suggesting that they derived from a common ancestral gene [50]. The most studied member of the PAT family is PLIN, a protein that regulates the access of lipases to neutral lipids in the core of LDs, hence controlling lipid homeostasis. Consistently, elevated basal lipolysis levels were observed in adipocytes obtained from PLIN knockout mice models [51].

LDs also host many other proteins-related to lipid homeostasis. These proteins can be classified according to their functions, including lipid biogenesis (long chain fatty acid CoA ligases, lanosterol synthase, squalene epoxidase), maintainance of intracellular lipid metabolism (long chain fatty acid CoA ligases) and lipid degradation (patatin-like phospholipase domain containing protein 2 (PNPLA2), CGI-58) [52].

In addition to PAT proteins and lipid homeostasis-related proteins in LDs, there are several groups of proteins that are called ‘refugee proteins’ which seem not to be relevant to the known functions of LDs [53]. These proteins are categorized as signaling proteins, membrane trafficking proteins, chaperons and proteins-associated with cellular organelles (Table 1). Signaling-related proteins are classes of proteins that accumulate on the surface of LDs. Major signalling proteins such as, mitogen-activated protein kinase (MAPK), phosphatidylinositol 3-kinase (PI3K) and Lyn proteins have been shown to localize on LD surfaces [54, 55]. Moreover, caveolins are another group of LD proteins that specifically form a coat by making invaginations and surrounding cellular membranes called caveolae. Caveolae function in endocytosis, signal transduction, cholesterol transport and growth control [33, 56, 57]. Caveolin-1 and caveolin-2 proteins were shown to reside on the surface of LDs. They generate membrane domains where they function as regulators of signaling proteins [58, 59]. Discovery of membrane trafficking proteins on the surface of LDs and the fact that interact with other cellular compartments through intarcellular motility, suggest that LDs are distinct organelles. Membrane trafficking-related proteins consist of five subgroup proteins: Small GTPases governing vesicle formation and motility; motor proteins such as kinesin and myosin that carry LDs on the cytoskeleton; soluble NSF attachment receptor (SNARE) proteins mediate membrane docking and fusion on LDs; vesicular traffic proteins (such as ARFs and COPs) that regulate cargo sorting and vesicle budding; and other membrane trafficking proteins of miscellaneous functions [4].

### LD Biogenesis

LD biogenesis is stimulated upon an increase in intracellular free FA levels. In order to prevent lipotoxicity, excessive free fatty acids are converted into neutral lipids and stored in cytosolic LDs. However, recent studies suggested that LDs are not only cytoplasmic but also can be found in the nuclei [60, 61]. Although the morphology of these ‘nuclear LDs’ is similar to their cytoplasmic counterparts, their biogenesis mechanisms remain unexplained. Nuclear LDs are thought to regulate nuclear lipid homeostasis and modulate signaling through lipid molecules [3].

**Fig. 2:**
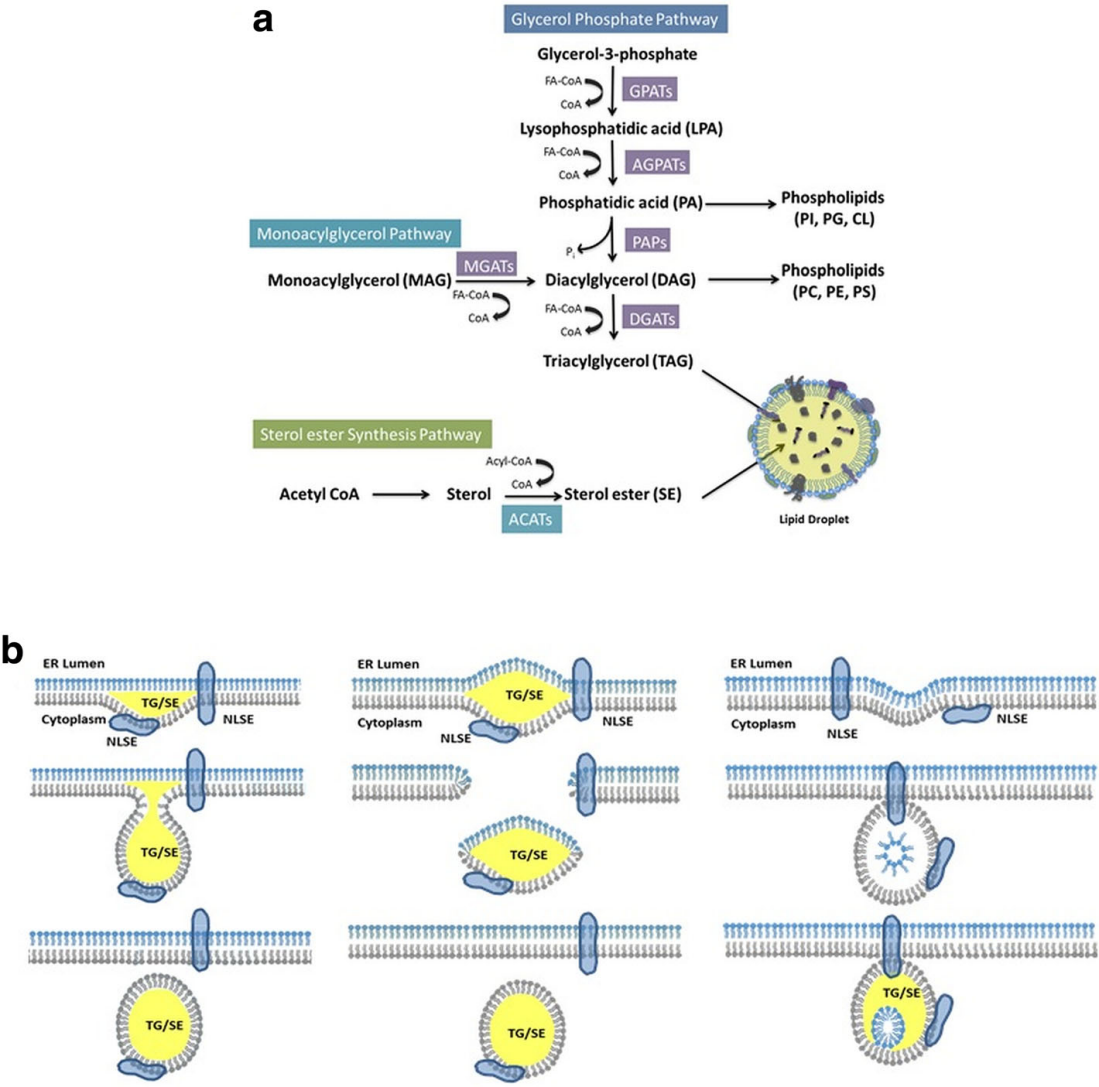
Neutral Lipid Synthesis, Lipid Droplet (LD) Formation and Growth. **a** Metabolic pathway of triglyceride (TG) and sterol ester (SE) synthesis, **b** LD Formation from Endoplasmic Retikulum (ER) by Neutral Lipid Synthesis Enzymes (NLSE). Left panel shows budding model of LDs from ER; middle panel shows bicelle formation model of LDs originating from ER; right panel shows hatching model of LDs from ER

Due to the unique hydrophobic/hydrophilic (amphipathic) structure of LDs, mechanisms of biogenesis of this organelle in the cell attracted much attention and several hypotheses were proposed [62–64]. Being different from most self-replicating organelles, LDs are mainly formed de novo. In addition, LDs could be derived via fission of pre-existing LDs [65]. In prokaryotes, neutral lipid accumulation seems to be initiated at specific lipid domains of plasma membrane and ends in the formation of cytoplasmic LDs [62]. On the other hand, as a eukaryotic LD biogenesis mechanism, there is evidence that LD formation occurs within the leaflets of ER phospholipid bilayer in discrete steps involving neutral lipid synthesis, progressive neutral lipid accumulation in the ER and cytosolic droplet formation. Increased volume of accumulated neutral lipid between the ER bilayer exceeds the solubility limits and LDs are thought to be ‘oiled out’ from the ER bilayer [66]. Consistent with the hypothesis, many electron microscopy studies in various cell types showed that cytosolic LDs are tightly associated with ER [67, 68] (Fig. 2).

#### Neutral lipid synthesis

Neutral lipid synthesis is regulated by complex pathways involving lipid metabolism enzymes and structural proteins that are permanently or transiently located to the ER and LDs. The first step of neutral lipid synthesis requires free FA activation. As free FA are chemically inert, their activation step is critical for LD biogenesis pathway. In mammalian cells, acyl-coA synthetase (ACS) enzymes activate long chain FAs to acyl-coA by esterifying with coenzyme A (CoA) (Fig. 2a) [69]. De novo TAG synthesis occurs in a four-step pathway involving glycerol-3-phosphate O-acyltransferase (GPAT), 1-acylglycerol-3-phosphate O-acyltransferase (AGPAT), phosphatidic acid phosphatase (PAP) (or lipin), and diacylglycerol acyltransferase (DGAT) enzymes. At the last step of the pathway, FAs, firstly activated to acyl-CoA, are converted to TAGs through DGAT1 and DGAT2 enzymes in mammalian cells. In addition, some LDs store mainly sterol esters (SE) specifically in macrophages, adrenocortical cells, ovarian and testicular interstitial cells. The synthesis of SE is conducted by acyl-CoA cholesterol O-acyltransferases (ACAT1 and ACAT2). The neutral lipid biosynthesis enzymes DGAT1, ACAT1, and ACAT2 were shown to localize on ER domains in mammalian cells [70–72].

#### LD Formation from ER

As intracellular FA promote LD formation, synthesized neutral lipids form a ‘lens’ between the leaflets of ER bilayer. Biophysical and in silico predictions suggest that, when TG holding limit of bilayer membranes is reached [23], the lens between ER bilayers is ‘oiled out’ and leads to the formation of nascent LDs. However, it is still an open question how phospholipid monolayer membrane of LDs is derived from ER bilayer membranes. Walther et al. claimed two models about LDs leaving ER membranes (Fig. 2b) [10]. The most widely accepted model states that neutral lipid lens buds out from the ER together with the outer leaflet of the bilayer membrane. This process is thought to be drived by structural LD associated proteins such as PAT proteins in a way that PAT proteins could mediate budding at specific domains of ER bilayer membrane [10]. Their suggested second model states that LDs are excised from both leaflets of the ER membrane bilayer as a bicelle (a lipid monolayer vesicle formed by the fusion of the tips of the outer and inner membranes of the ER with accumulated lipid lens within) [10]. In addition to these models, Fujimoto et al. suggested that LDs are excised from ‘both leaflets of the ER membrane bilayer as a bicelle’ by hatching mechanism (bilayer budding and monolayer membrane formation because of micelle-like isolation of the inner membrane leaflet in the LD lumen) (Fig. 2b) [22]. Although neither of these models rely on solid data, they could hypothetically explain how hydrophobic cores of LDs are surrounded by phospholipid monolayer membranes that are derived from ER membranes and how some ER membrane proteins are targeted to newly formed LDs.

#### Growth of lipid droplets: the size matters

Once LDs are synthesized, they typically keep growing because of the excessive amount of intracellular FA in cells and reach a final size. The size of LDs varies within a wide range (0.4–100 μm) in different cell types [73]. Even in the same cell, the size of LDs may dramatically differ under changing pathophysiological conditions. Recent studies have shown that subpopulations of LDs may differ in terms of size, morphology, and function within the same cell [74, 75]. Many proteins (e.g. Fsp27, seipin, FITM2 and perilipin1) and lipid factors (e.g. phosphatidylcholine and phosphatidic acid) have been shown to be involved in LD growth mechanisms [73].

One possible mechanism is growth of LDs through fusion of highly mobile smaller LDs [76, 77]. Although there is conspicuous skepticism in the literature, LD fusion rates are high enough to allow LD growth [78], RNAi knockdown of fusion mediator SNARE proteins prominently decreased fusion rates and size of LDs [76]. In addition, mutant form of the yeast homologue of seipin in the mammals regulates phosphatidic acid metabolism and leads to ‘supersized LDs’ by enhancing coalescence of them [31]. Similarly, knockdown of phosphocholine cytidylyltransferase (CCT) enzymes, catalyzing the rate limiting step of phosphatidylcholine synthesis, dramatically induce fusion of LDs, balance surface volume ratio and regulate LD size [79]. Another possible mechanism for growth of droplets is the lateral transfer of synthesized neutral lipids from ER to LDs. If nascent LDs remain attached to ER, as neutral lipid synthesis enzymes on the bilayer membrane of the ER convert FA to neutral lipids, these molecule may be transferred to adjacent LDs [1]. Similar to this mechanism, Gong et al. stated that lipid droplet growth occurs by lipid transfer at LD contact sites between adjacent LDs via Fsp27 (Fat specific protein-27 or cell death-inducing DFF45-like effector C (Cidec)) protein [80]. Ectopic expression of Fsp27 protein promotes formation of unilocular large LDs through stimulation of TG accumulation [80–82]. Alternatively, Fujimoto et al. suggested that neutral lipid synthesis occurs on the surface of LDs [83]. Since phospholipid monolayers of LDs are derived from ER membranes, neutral lipid synthesis enzymes originating from these membranes could be directly transfered to the surface of LDs. Particularly, during lipid loading, DGAT2 enzymes which normally localize to the ER, were shown to localize on the surface of LDs [71, 84]. However, it is ambiguous whether earlier steps of neutral lipid synthesis also take place on the surface of LDs.

### Function of LDs other then lipid storage

Interestingly, LDs were involved in various pathological events and innate immunity [85]. LDs were shown to be targeted by various pathogens including viruses, bacteria and parasites. Hepatitis C virus (HCV) was appeared to use the LDs for proliferation and assembly of capsid proteins were shown to take place in the vicinity of host LDs [86]. In addition to HCV, many viruses including GB virus B and dengue virus (DENV) also hijack LDs for viral replication [87, 88]. As LDs may also be related to the pathogenesis of hepatic steatosis caused by viral infections and from this perspective, LDs may become a theurapetic target [22]. Furthermore, LDs may serve in the cytoplasmic sequesteration of proteins such as histones and α-synuclein [37, 52]. In *Drosophila* embryos, histones are found in high abundance on the surface of LDs and they are rapidly transferred to the nucleus when needed [89]. Recently, histone storage function of LDs was also defined as a defense mechanism against pathogens. Indeed, histones around LDs were shown to possess bactericidal activity [3]. Neuropathological protein aggregates of α-synuclein and amyloid β peptide were shown to be in a close proximity with cytosolic LDs in various cell types [90–92]. In addition, recently Moldavski et al. stated that LDs play important roles as clearing mechanisms of inclusion bodies in cells [93]. Protein sequestration role of LDs may explain the presence of ‘refugee proteins’ that are found associated with LDs, and provide a dynamic regulatory mechanism for protein storage and degradation [53].

### The catabolic pathways of LDs

As the LDs reside at the center of cellular lipid and energy homeostasis, their catabolism is strictly under the control of hormones and activation of enzymes. So far, LDs are known to break down mostly by lipolysis, however, recent discoveries that showed a molecular connection between lipolysis and autophagy mechanisms led to the identification of the involvement of selective autophagic forms, lipophagy and CMA, in LD catabolism.

#### Lipolytic hydrolysis

**Fig. 3:**
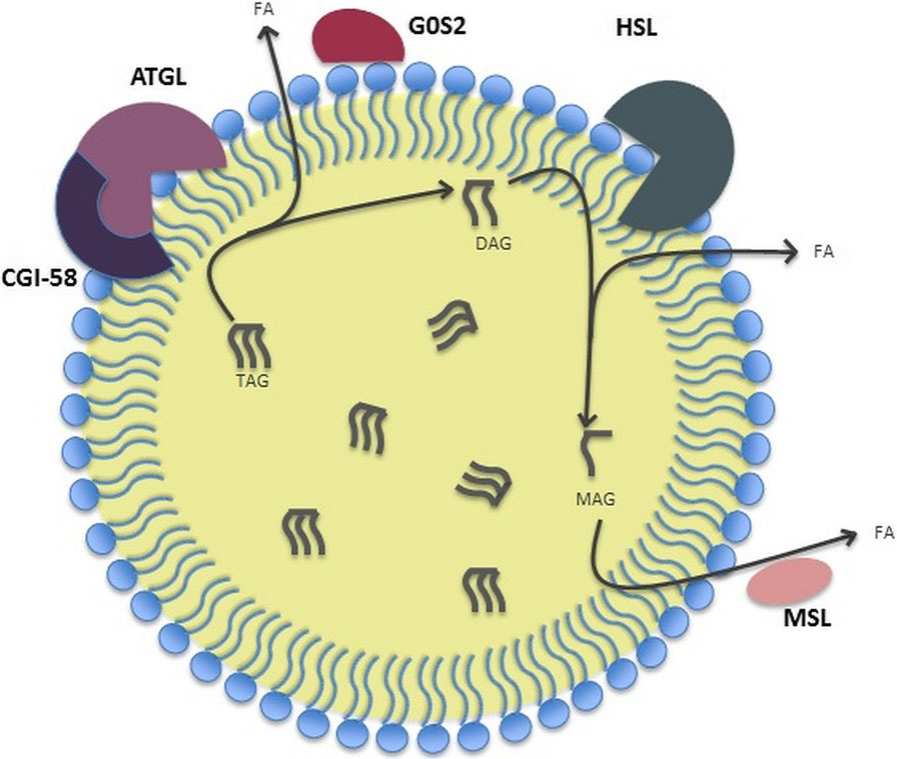
Lipolysis of Lipid Droplets

Under fed conditions, LDs store TAGs mainly in adipose tissues and hydrolysis of ester bonds between long chain FAs and glycerol backbone in TAGs is called “lipolysis”. During the first step of lipolysis, protein kinase A (PKA) phosphorylates one of the PAT protein family member, PLIN1, and leads to its proteasomal degradation [94]. This, results in the release of ATGL activator protein CGI-58, which then initiates TAG breakdown via activated ATGL [95, 96]. ATGL selectively catalyzes the first step of TAG hydrolysis to generate diacylglycerols (DAGs) and free FAs [97]. The second step of lipolysis is depend on the activation of hormone sensitive lipase (HSL) which is a multifunctional enzyme that is capable of hydrolyzing both the first and the second step of lipolysis. HSL hydrolyzes DAGs and produce monoacylglycerol (MAG) and FAs [98]. Within the lipolysis cascade, HSL functions as a rate-limiting enzyme for DAG catabolism [99, 100]. In the last step of lipolysis, MAGs are released into the cytosol and eventually cleaved by MGL to generate glycerol and FA [101] (Fig. 3).

Under nutrient deprivation, products of lipolysis are secreted from the adipose tissue to the periphery via bloodstream, which are then used for β-oxidation and ATP production. In contrast, in non-adipose tissues, mitochondria or peroxisomes can directly oxidize products of lipolysis through β-oxidation and release acetyl-CoA [102].

#### Lipophagy

**Fig. 4:**
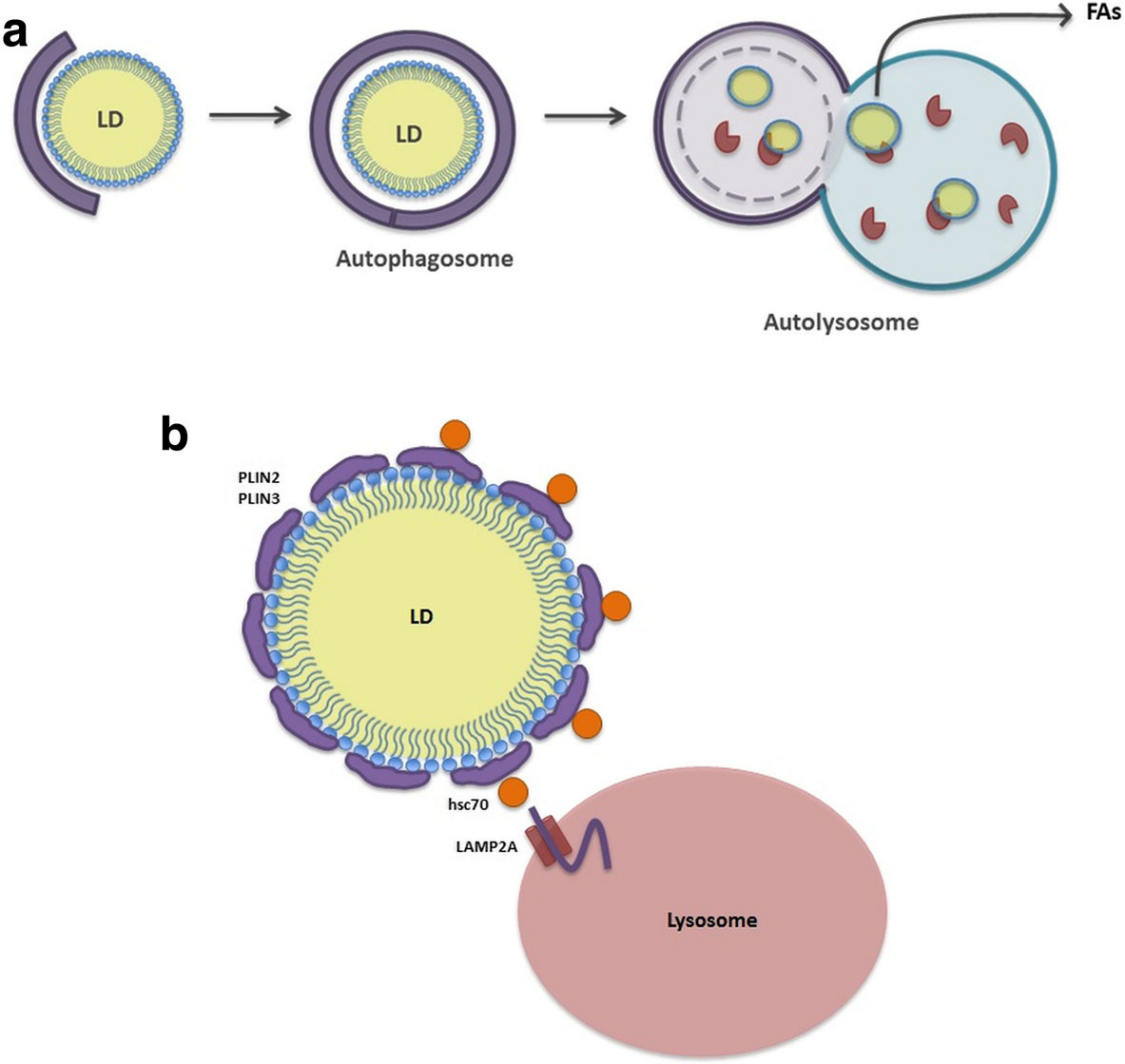
Lipid Droplet Degradation by Autophagy Mechanisms. **a** Lipophagy **b** Chaperone Mediated Autophagy (CMA)-dependent degradation

Lipophagy was first described in mouse hepatocytes under starvation [103]. It was shown that LDs were mobilized in order to generate free FAs. Pharmacological or genetic inhibiton of lipophagy resulted in elevated TAG concentrations, increasing concomitantly LD numbers and size. Electron microscopy data confirmed sequesteration of LDs by double-membraned autophagosomes that labeled positive with the autophagy marker LC3 (Microtubule-Associated Proteins 1A and 1B, Light Chain 3). Intriguingly, LC3 as well as PLIN1 and PLIN2 proteins were found in LDs and lysosomes that were isolated from starved livers. In addition, LDs were shown to colocalize with lysosomes, indicating a role for lysosomes in lipid turnover via LDs break down [103]. LDs were shown to be selectively engulfed by a autophagosomes that was followed by the fusion of autophagosomes with lysosomes (autolysosomes) for degradation. Therefore, lipophagy is an LD-selective type of macroautophagy. In line with that, inhibition of the small GTPase dynamin-2 caused the accumulation of LDs in autolysosomes [104] (Fig. 4a).

Substrate labeling is a general mechanism used by cellular degradation machineries. So, how does lipophagic machinery preferentially recognize LDs as substrates? Proteomic analyses of LDs from different organisms showed that membrane GTPase Rab7 is associated with LDs and suggested a universal role of Rab7 in the regulation of LD docking and degradation [105]. Involvement of GTPases in endosome-autophagosome interactions [106] is made Rab7 a strong candidate for selection of LDs as lipophagic substrate. Activation of lipophagy in starved hepatocellular cells lead to significant increase in Rab7-LD association while Rab7 mutants, defective in binding to Rab7-interacting lysosomal protein (RILP) failed to promote lipophagy [18]. Furthermore, starvation-activated Rab7 was shown to lead to the recruitment of multivesicular bodies to the LD-autophagosome complex and induced the formation of amphisomes through fusion of autophagosomes and endosomes [18]. In this study, microscopy data revealed an interaction between LDs, autophagosomes and lysosomes, which is consistent with the concept that LDs do not fuse directly with lysosomes [103]. In addition to hepatocytes, the essential role of Rab7 for autolysosome-mediated LD degradation during hormone-stimulated lipophagy was also shown in adipocyte cells. In these cells, LD-associated PLIN1 protein inhibited lipophagy by blocking Rab7 binding to the LD surface [18]. Consequently, Rab7 was identified as a protein regulating LD docking via its effector protein RILP and FYVE and coiled-coil domains containing protein 1 (FYCO1) that directly connects the cargo to LC3 proteins on autophagosome membranes [18].

In adipocytes, the membrane curvature protein Bif-1 was found as a novel regulator protein for lipophagy [107]. Induction of lipophagy has been shown to promote Bif-1-dependent degradation of LD-associated PLIN1, and deficiency of this protein led to decreased TAG hydrolysis, indicating that Bif-1 is essential for lipophagy-dependent PLIN1 degradation, and thereby LD break down [107]. Note that, membrane curvature varies according to LD size, however, whether Bif-1 affects LD targeting by lipophagy through its effects on LD size, is still unknown.

Although the role of lipophagy in LD degradation was first described in hepatocytes [103, 108], several recent studies shown activation of this mechanism in various cell types, such as neurons [109, 110], glial cells [109], foam cells [111], enterocytes [112], T cells [113], fibroblasts, adipocytes [114] and prostate carcinoma cells [115]. Moreover, lipophagy was demonstrated in yeast [116], *C. elegans* [117] and some fungus species [118]. Indeed, mechanisms that trigger lipophagy may vary in each cell type, and it could be context-dependent. It is likely that cells adapted lipophagy mechanisms in order to better deal with extreme conditons, such as lipotoxicity, nutrient deprivation etc. However, the core components that are essential for lipophagy seem to be conserved in most cell types.

#### Chaperone-mediated autophagy (CMA)

More recently, involvement of another selective type of autophagy, CMA, in degradation of LD-associated PLINs proteins, PLIN2 and PLIN3 was described [19]. In this context, activation of CMA was shown to induce PLIN2 and PLIN3 turnover, which caused recruitment of ATGL and lipophagic protein ATG, leading to the degradation of LDs. Consistantly, inhibiton of PLINs degradation in CMA-deficient model systems, resulted in both reduced ATGL recruitment, and lipophagy activation gave rise to LD accumulation. Therefore, functional CMA was shown to be essential for the removal of LD-associated proteins, which in turn led to the accumulation of lipases and lipophagy proteins on specific regions of LDs [19] (Fig. 4b).

#### Cross-talk between lipolysis and lipophagy/CMA mechanisms

Even though basic molecular mechanisms of lipolysis, lipophagy and CMA are different, recent investigations indicate that these independent pathways actually cross-talk. For example, ATG2A, an essential autophagy protein was also found on LDs, and genetic knock-down of ATG2A and ATG2B resulted in defective lipophagy as well as LD accumulation [119]. A recent study with starved mouse embryonic fibroblasts (MEF) demonstrated that FAs derived from LDs end-up in mitochondria which is required only for ATGL-dependent lipolysis activation not for lipophagy [120]. In the same study, size and number of LDs increased as result of autophagic degradation of other cellular membrane components, confirming that overall lipid content that recycled via autophagy, contributed to the growth of LDs. As mentioned before, Bif-1 regulating curvature of various membranes including autophagic membranes, was identified as a protein involved in lipophagy regulation in adipose tissues [107]. PLIN2 and PLIN3 are CMA substrates, and they contribute to the cross-talk between lipolysis and CMA. Promotion of ATGL and lipophagy proteins by CMA activation was shown to trigger not only lipophagy but also lipolysis, indicating that PLIN2 and PLIN3 undergo CMA-mediated degradation before the initiation of lipolysis [19]. Moreover, lipolysis has been shown to depend on PLIN2 phosphorylation by AMPK, which occured after the interaction of PLIN2 with CMA chaperone protein Hsc70 [20]. A complex cross-talk between lipolysis and lipophagy has been described in brown adipose tissue, in which both central nervous system (CNS) and local cytosolic lipases were involved. Here, the autophagosome marker LC3 recruited ATGL and HSL to LDs, supporting coordinated regulation of lipolysis by both CNS and peripheral protein-protein interactions [121].

On the other hand, the existance of a reverse relationship between lipolysis and lipophagy has also been shown in which lipolysis-dependent LDs breakdown regulated autophagosome formation. LDs was shown in close proximity of autophagosomes with transient ‘kiss and run’ interactions contributing to autophagosome biogenesis [122]. In this process, patatin-like phospholipase domain containing protein-5 (PNPLA5), a lipase localized on the surface of LDs, was shown to be needed for optimal initiation of autophagosome formation by generating DAG, a building block for phospholipid constituents of the double-membrane structure [122]. Consistent with this, additional evidence has been provided by a yeast study, demonstrating that deletion of TAG and SE synthesis enzymes resulted in the inhibition lipophagy [123]. In a very recent yeast study, absence of LDs caused morphological changes in the ER due to defective FA synthesis, compromised autophagosome biogenesis [124]. Therefore, active lipolysis on the LD surface or LD-ER contact sites is required for the de novo formation of autophagosomes [122–124].

### Pathophysiological relevance of lipolysis, lipophagy and CMA

As LDs play central role in the regulation of intracellular lipid and energy metabolism, abnormalities of LD-related mechanisms, including lipolysis, lipophagy and CMA are involved in many pathological and physiological conditions. For example, neutral lipid storage disease (NLSD), atherosclerosis, obesity etc. [8, 125].

#### Neutral lipid storage disease (NLSD)

Neutral Lipid Storage Disease (NLSD) is a heterogenous group of rare autosomal recessive disorder characterized by abnormal accumulation of LDs in multiple tissues and lymphocytes. Mutations in one of the two genes coding for ATGL or CGI-58 (proteins located on the surface of LDs) are associated with the NLSD [126, 127]. Both ATGL and CGI-58 coordinately function in the lipolysis of LDs; however, mutations in each gene result in different symptoms. Mutations in CGI-58 (a coactivator of ATGL), are associated with NLSD with ichthyosis known as Chanarin-Dorfmann syndrome (CDS) characterized by defective permeability barrier of the skin [128–130]; whereas, ATGL mutations lead to severe NLSD with cardiac myopathy (NLDSM) [131]. These symptomatic differences may be related to the different functions of CGI-58 independent from ATGL. For example, CGI-58 was shown to have acyltransferase activity in phosphatidic acid biosynthesis and play an ATGL-independent role in the phospholipid metabolism [132]. It was suggested that, apart from its lipolytic activity, CGI-58 may facilitate the utilization of hydrolysis products of TGs as phospholipids, and maintain TG homeostasis [132].

#### Obesity

Obesity is a common health problem, which is characterized by excessive lipid storage in various cell types such as adipocytes, muscle cells and hepatocytes. Different types and degrees of obesity were shown to be directly related with the lipophagic activity of cells and the size of their lipid storages [133]. Additionally, lipophagic activity was found to be upregulated in the adipose tissue of obese people, and especially in those who have particularly high intra-abdominal fat accumulation. In these people, visceral lipid distribution or hypertrophic adipocytes could also be seen concomitantly with insulin resistance [134]. Under these conditons, upregulation of lipophagy might protect cells against lipotoxicity by clearing excessive intracellular lipids, and decrease obesity-dependent mortaility in patients [134]. In fact, the amplitude of lipophagic activation may depend on the metabolic properties of individuals. For example, in patients diagnosed with type-2 diabetes which is characterized by insulin resistance, inhibition of the main energy regulator pathway, mTOR, was described as the responsible mechanism for lipophagic upregulation [135]. Yet, enhanced LD formation can sometimes lead to excessive release of FAs from LDs, and lipophagy may favor intracellular lipotoxicity under these conditons [135].

Accumulation of LDs in tissues in high amounts may cause chronic inflammation which is identified as one of the hallmarks of obesity-related metabolic disorders. Indeed, enlarged LDs in the adipose tissue may lead to cellular remodeling, and finally lead to macrophage-driven chronic inflammation [136]. Activation of macrophages is a energy-dependent mechanism and relies on FA oxidation, suggesting a possible involvement of lipophagy in this mechanism. Accordingly, lipophagy was shown to increase with obesity in adipose tissue macrophages; however, their activation was not affected, suggesting that lipophagy only regulates elavated intracellular lipid level in the adipose tissue [114]. Liu et al. have shown decreased level of lipophagy with obesity in hepatic macrophages, and inhibiton of lipophagy was shown to favor the immune responses by promoting proinflammatory macrophage activation [137]. Very recently, lipophagy was found as an essential catabolic mechanism in adipose tissue macrophages, and it increased with obesity. But intriguingly, genetic or pharmacological inhibition of lipophagy did not change lipid balance, indicating existence of another pathway critical to lysosome TG hydrolysis [138]. All these data indicate that the role of lipophagy in macrophage-driven inflammation may depend on metabolic conditions of obesity or inflammatory stimulus.

#### Fatty liver diseases

The important role of lipophagy regarding LD breakdown in hepatocytes suggests that impaired lipophagy may contribute to the development of steatotic liver diseases such as non-alcoholic and alcoholic steatohepatitis [133]. These diseases are characterized by increased level of lipid storage in LDs, and they progressively lead to the development of chronic liver injury and its complications, such as fibrosis and hepatocellular cancer [139, 140].

Promotion of lipophagy by resveratrol treatment was shown to attenuate methionine choline-induced non-alcoholic steatohepatitis [141]. Consistently, in a recent study, lipophagy has been found to play an important protective role in methionine choline-induced non-alcoholic steatohepatitis [142]. Lipophagy has also an important protective role in acute alcohol-induced hepatic steatosis and injury [143]. Likewise, impairment of both non-selective macroautophagy and selective CMA promoted oxidant-induced liver injury, indicating that oxidative stress resistance and liver injury is connected to autophagic activity [144]. Another report also support the protective role of lipophagy against chronic alcohol-induced hepatotoxicity caused by oxidative stress [145].

Liver fibrosis may be the first step of more serious cirrhotic liver conditions. Hepatic stellate cells (HSCs) activation for the development of this condition, and lipophagy plays a role as well [146, 147]. HSCs are quiescent cells having large lipid stores that are metabolized during activation. Increased lipophagy was shown in HSCs upon fibrotic stimuli and HSC-specific inhibiton of lipophagy blocked their activation, leading to improved liver fibrosis in mice and in human tissues [147]. On the other hand, controversy exist over the effect of lipophagy modulation in this disease. For example, pharmacological induction of lipophagy was shown to be a promissing treatment for liver fibrosis in alpha-1 antitrypsin deficiency [148]. Even though HSCs activation is a common phenomenon for fibrogenesis among tissues, whether lipophagy mediates fibrosis in other organs is still unknown.

Today, it is well known that after many years of chronic non-alcoholic or alcoholic steatotic liver diseases or hepatitis virus infection hepatocellular carcinoma (HCC) might develop. Hepatitis B virus (HBV) and hepatitis C virus (HCV) replication was shown to be induced by autophagy activation both in vitro and in vivo studies [149, 150]. A number of reviews have described the role of non-selective and/or selective autophagy in HCC, and accumulating data indicate changes of autophagic responses in this disease [133, 151]. However, there are discrepancies among studies that were published so far, and development of autophagy-based novel therapeutic strategies for HCC require further studies.

#### Cholesterol ester storage disease (CESD)

Cholesterol ester storage disease (CESD) is an autosomal recessive genetic disease caused by the mutation of the *LIPA* gene encoding lysosomal acid lipases (LAL). Deficient LAL activity lead to insufficient lipolysis and eventually intracellular accumulation of cholesterol ester and TGs [152]. The most severe form of CESD is identified as Wolman Disease that mostly presents in infancy and mainly result in infant fatality. Affected cells are characterized by the presence of lysosomes that are filled with excessive LDs and cholesterol clumps [153]. The involvement of lipophagy was shown in a study analyzed LD mobilization in macrophages [111]. LDs are the major organelles for cholesterol storage and the study demonstrated that cholesterol efflux from macrophage foam cells was regulated by lipophagy, which rely on LAL function. In line with these results, genetic knock-down of lipophagy in macrophages or knockout in the whole mice resulted in insufficient clearance of cholesterol in cells and tissues [111].

#### Atherosclerosis

Accumulation of cholesterol in macrophage foam cells also contribute to another common disease, atherosclerosis. The main cause of the disease is the accumulation of excessive cholesterol in arterial walls eventually leading to heart failure, stroke and cardiac dysfunction. Excessive cholesterol is esterified by ACAT enzymes and stored as cholesterolester (CE) in LDs of macrophages. Studies revealed that LD-associated proteins ACAT1 and ABCA1 regulating cholesterol esterification play crucial roles in atherosclerosis. In hyperlipidemic mice, deletion of ACAT1 results in both impairment of ABCA1 dependent cholesterol efflux and elevated atherosclerosis [154]. Moreover, among the PAT protein family of LDs, adipose differentiation-related protein (ADFP) was strongly associated with foam cell formation and atherosclerosis [155, 156].

The importance of lipophagic activation in controlling intracellular LDs level and the recent finding that lipophagy contribute LD mobilization in macrophages provides a new point of view in the pathogenesis of atherosclerosis. In addition to the dysfunction of above mentioned LD-associated proteins, it is likely that defective lipophagy may compromise the chronic exposure to excessive circulating lipids of the artery wall macrophages and may result in their remodelling into “foam cells”. However, the significance of insufficient lipophagy underlying foam cell formation or progression is still not known. On the other hand, in a recent study on the tumor-supressor gene programmed cell death 4 (PDCD4), it was demonstrated that the protein inhibited autophagy in macrophages, and *Pdcd4* knockout mice displayed increased atherosclerosis, indicating a link between autophagy and atherogenesis [157].

#### Lipodystrophies

Apart from excessive accumulation of lipids, deficiency of LDs in cells may lead to lipodystrophies. Particularly, defects in the synthesis of neutral lipids lead to deficiencies in LD formation and lead to the formation of abnormal or degenerative white adipose tissue. Genetic causes of lipodystrophies include various genes encoding proteins related to LD synthesis, storage and regulation such as acylglycerol-phosphate acyltransferase (*AGPAT2)*, seipin (*BCSL2)* and caveolin (*CAV1)*. In addition, lamin A/C (*LMNA)*, peroxisome proliferator-activated receptor-γ (*PPARG)*, Akt2/protein kinase B (*AKT2)*, and endoprotease Face-1 (*ZMPSTE24)* were identified as defective genes in partial lipodystrophies [158]. Insufficient LD biosynthesis in adipose tissue often leads to massive hepatic steatosis with subsequent metabolic abnormalities, including insulin resistance, diabetes, and hypertension [159].

## Conclusion

In recent years, except the neutral lipid storage function of LDs, understanding of their various metabolic roles and intracellular interactions with many compartments makes this field as an attractive research area. Novel findings in selective types of autophagy, lipophagy and CMA, also contributed to a better of understanding of intracellular catabolic pathways regulating LDs. More importantly, signals that madiate the crosstalk between lipolysis and lipophagy lead to the consideration of these mechanisms as promising therapeutic targets. However there are a large number of questions that still need to be answered. Particularly, the pathophysiological relevance of LDs and their catabolic mechanisms must be further examined in order to discover potential therapies for common metabolic diseases such as obesity or atherosclerosis.

## Abbreviations

ACS: Acyl-coA synthetase
ADFP: Adipose differentiation-related protein
ADRP: Adipocyte differentiation-related protein
AGPAT: 1-acylglycerol-3-phosphate O-acyltransferase
AKT2: Akt2/protein kinase B
ATGL: Adipose triglyceride lipase
BCSL2: Seipin
CAV1: Caveolin
CCT: Phosphocholine cytidylyltransferase
CESD: Cholesterol ester storage disease
CGI-58: Comperative gene identification-58
Cidec: Cell death-inducing DFF45-like effector C
CMA: Chaperone mediated autophagy
DAG: Diacylglycerols
DENV: Dengue virus
DGAT: diacylglycerol acyltransferase
ER: Endoplasmic reticulum
Fsp27: Fat specific protein-27
FYCO1: FYVE and coiled-coil domains containing protein 1
GPAT: Glycerol-3-phosphate O-acyltransferase
HBV: Hepatitis B virus
HCC: Hepatocellular carcinoma
HCV: Hepatitis C virus
Hsc70: Heat shock cognate 70
HSL: Hormone sensitive lipase
LAL: Lysosomal acid lipases
LAMP2a: Lysosomal-associated protein 2a
LC3: Microtubule-Associated Proteins 1A and 1B, Light Chain 3
LD: Lipid droplet
LMNA: Lamin A/C
MAG: Monoacylglycerol
MAPK: Mitogen-activated protein kinase
MEF: Mouse embryonic fibroblasts
MGL: Monoglyceride lipase
NLDS: Neutral lipid storage disease
NLDSM: NLSD with cardiac myopathy
PAP: phosphatidic acid phosphatase
PC: Phosphatidylcholine
PDCD4: Programmed cell death 4
PE: Phosphatidylethanolamine
PI: Phosphatidylinositol
PI3K: Phosphatidylinositol 3-kinase
PKA: Protein kinase A
PLIN: Perilipin
PNPLA2: Patatin-like phospholipase domain containing protein 2
PPARG: peroxisome proliferator-activated receptor-γ
PS: Phosphatidylserine
RILP: Rab7-interacting lysosomal protein
SE: Sterol ester
SM: Sphingomyelin
SNARE: Soluble NSF attachment receptor
TAG: Triacylglyceride
TIP47: Tail-interacting protein of 47 kDa
ZMPSTE24: Endoprotease Face-1
